# Comparison of the initial ovarian response, the synchrony of oestrus and ovulation and chronic stress response after administration of 100 or 250 μg of GnRH to randomly cycling *Bos indicus* cattle

**DOI:** 10.1111/avj.13196

**Published:** 2022-06-28

**Authors:** M Abdallah, C Joone, S Edwards, S Das, J Cavalieri

**Affiliations:** ^1^ College of Public Health, Medical and Veterinary Sciences James Cook University Townsville Queensland 4811 Australia; ^2^ Vetoquinol pharmaceuticals, L2/485 Kingsford Smith Dr Hamilton Queensland 4007 Australia; ^3^ College of Science and Engineering, James Cook University Townsville Queensland 4811 Australia

**Keywords:** *Bos indicus*, oestrous synchronisation cortisol GnRH stress

## Abstract

**Objective:**

This study investigated the effects of administering saline, 100 or 250 μg of gonadotrophin releasing hormone (GnRH) on ovarian response, synchrony of oestrus and ovulation and chronic stress response in *Bos indicus* cattle.

**Design:**

Randomised control.

**Methods:**

Animals were either left untreated (n = 20) or on day 0 treated with an intravaginal progesterone releasing device and either saline (n = 24), 100 μg (n = 35), or 250 (n = 35) μg of GnRH, intramuscular (IM). Blood was sampled 1.4 h after administration of treatment to monitor concentrations of luteinising hormone (LH) and P4 in serum and again 5 days later. On day 5 intravaginal P4 releasing device were removed, cloprostenol was administered IM and again 8 h later. Oestrus and ovulation were then monitored with ultrasonography for 6.5 days. Hair was clipped on day 55 for analysis of hair cortisol concentrations (HCC).

**Results:**

No significant differences were found between Saline and GnRH treatments in the odds of inducing a new corpus luteum (CL) and the synchrony of oestrus or ovulation. HCC did not differ significantly between treatments. Mean concentrations of LH in serum on day 0 were less in the Saline compared to 100 and 250 μg GnRH treatments but did not differ between different doses of GnRH.

**Conclusion:**

Mean concentrations of LH and the odds of inducing a new CL were not increased after administering 250 μg compared to 100 μg of GnRH. Animal handling events in the study did not influence HCC. Further research is needed to better optimise responses to GnRH in *B*. *indicus* cattle.

**Abbreviations**
AI: artificial insemination
CI: confidence interval
CL: corpus luteum
GnRH: gonadotrophin releasing hormone
HCC: hair cortisol concentration
IM: intramuscular
IVD: intravaginal P4 releasing device
LH: luteinising hormone
OR: odds ratio
P4: progesterone

Artificial insemination (AI) is used to improve genetic merit and productivity in beef cattle located in tropical and subtropical regions of the world.^1^ Two main types of treatment protocols are utilised in beef cattle herds that enable AI to be performed at set times. These involve administration of intravaginal progesterone (P4) releasing device (IVD) and either administration of esters of oestradiol or gonadotrophin‐releasing hormone (GnRH) to synchronise emergence of preovulatory follicles and to subsequently induce timed ovulation.^2^, ^3^ In this way, these protocols help control follicular development enabling more precise synchronisation of oestrus and ovulation in cattle.^4^

Options for synchronising preovulatory follicular development are limited, with the administration of oestradiol to beef and dairy cattle now prohibited in many countries.^5^ One factor that limits responses to GnRH‐based protocols is the percentage of animals that ovulate following administration of GnRH at the time of inserting an IVD. Ovulation rates in most studies have varied between 50 and 65% in dairy cows^4^, ^6^, ^7^, ^8^, ^9^, ^10^ and 33%–66% in beef cows^11^, ^12^, ^13^, ^14^ and heifers.^15^ Increasing the ovulatory response to the first treatment of GnRH as part of the Ovsynch protocol has been associated with improved synchronisation and pregnancy rates to AI in lactating dairy cows.^4^, ^8^, ^9^, ^16^ In addition, the use of a 5‐day, treatment with P4 after administration of GnRH has improved fertility in some studies.^17^, ^18^ Thus strategies to improve ovulation rates to treatment with GnRH in beef cattle while using a 5‐day treatment with P4 may improve fertility to a timed AI.

Administration of greater doses of GnRH has increased the magnitude of the luteinising hormone (LH)^9^, ^19^, ^20^ surge and the ovulatory response^9^ in *Bos taurus* cattle in some studies but there have been no studies that have examined effects in randomly cycling *Bos indicus* cattle in Australia. Elevated circulating concentrations of P4 at the time of administering GnRH can also reduce the magnitude of the induced LH surge in *B*. *taurus* cattle,^21^ which could suggest that a higher dose of GnRH at the time of insertion of an IVD could improve the ovulatory response.

The public is increasingly concerned about the welfare of animals that are used to produce products for human consumption. Administration of IVDs is frequently associated with vaginitis and a purulent vaginal exudate which, although self‐limiting, has been observed in 62%–77% of dairy cows^22^, ^23^, ^24^ and could have an impact on cow comfort during treatment. Administration of intravaginal inserts and associated treatments and monitoring of ovarian responses with repeated transrectal ultrasonography^25^ and interactions with humans^26^ is expected to cause a degree of stress in animals although there are very few reports that examine stress responses associated with treatments that synchronise oestrus in cattle. Concentrations of cortisol within hair samples provide an indicator for the assessment of stress in animals^27^ and could provide one measure of the degree of stress associated with treatments used to synchronise oestrus in cattle and experimental procedures used to investigate treatments.

The aims of this study were to compare the odds of inducing a new corpus luteum (CL) and synchrony of oestrus and ovulation following the administration of saline or two doses of GnRH to *B*. *indicus* heifers and cows and to determine the stress response by measuring hair cortisol concentrations (HCC). Our hypotheses were that, the administration of 250 μg compared to 100 μg of GnRH would increase the LH response and odds of inducing a new CL in *B*. *indicus* females and that the administration of treatments and monitoring of cattle during this study would result in a greater HCC compared to animals that were not treated and intensely monitored.

## Materials and methods

A randomised control study was conducted between November 2019 and January 2020 in *B*. *indicus* female beef cattle at the James Cook University's Tropical Veterinary Research Station, Fletcherview located northwest of Charters Towers, Queensland (20°04.603′S, 146°15.812′E). Ethics approval for this study was granted by the James Cook University Animal Ethics Committee (Approval number: A2589).

### Description of the herd

Animals enrolled in the study consisted of nulliparous *B*. *indicus* (Brahman) heifers about 22 months of age (n = 78) and mature, non‐lactating *B*. *indicus* (Brahman) cows 3–12 years of age (n = 36) that had normal, non‐pregnant reproductive tracts when examined with rectal palpation and transrectal ultrasonography. Cattle grazed a mixture of native and improved pasture supplemented with about 1.5 kg/day of molasses containing 8% urea and 5% soya bean meal. Cattle were also supplemented with Rhodes grass hay ad libitum from days 5 to 12 of the study.

### Sample size calculation

Sample size estimates were calculated using WINPEPI version 11.65.^28^ From a separate study, the percentage of *B*. *indicus* heifers ovulating in response to administration of 100 μg of GnRH was 26% (20/77).^29^ The number of experimental animals required to detect an increase in the initial ovulatory response from 26% to 65% in cows treated with 250 μg of GnRH was estimated to be 25 cows per group. A response rate of 65% was considered to be a minimal desired response rate. Statistical power was set at 0.80 and the significance level was set at 0.05.

### Treatments

**Figure 1:**
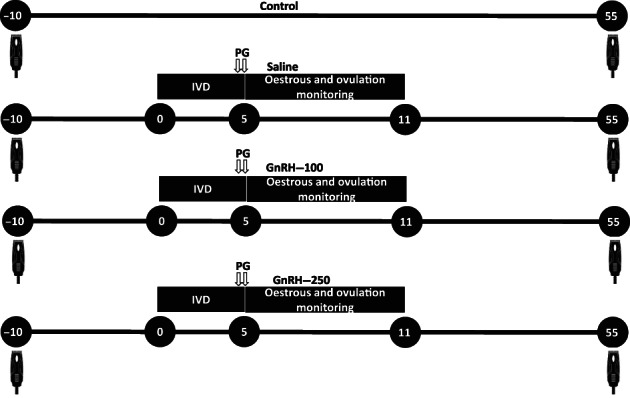
Diagrammatic outline of the experimental protocol used in the study. Hair was clipped in the lumbar region on day 10 from a random group of animals (n = 48). The untreated group received no further treatment. The saline, GnRH‐100 and GnRH‐ 250 groups had an intravaginal P4 releasing device (IVD) inserted on day 0 and were concurrently treated with either 1 mL of 0.9% saline, or 100 or 250 μg of GnRH IM, respectively. Cloprostenol (0.5 mg, PG) was injected at the time of removal of IVD on day 5 and again about 8 h later. Aids for the detection of oestrus were applied on day 5. Detection of oestrus and ovulation was undertaken from days 5 to 11.5. Blood samples were collected from a subset of animals on day 0, between 1 and 2 h after administering treatments to measure concentrations of P4 and LH and again on day 5 to measure concentrations of P4. Hair was again clipped from the same site from the same animals on day 55 and used to measure concentrations of cortisol.

At enrolment (day −10), cows from all groups were weighed and body condition scored (BCS) using a five‐point scale, where 1 = emaciated and 5 = obese. Hair was clipped from a random group of animals (n = 48) with electronic clippers from a region that was approximately 15 cm × 15 cm in size, just cranial to the sacrum. On day 0, cows were stratified by age (heifer or cow) and then randomly assigned to 1 of 4 treatments (Figure 1) while ensuring that at least 8 heifers and 4 cows in each treatment had been clipped. Cows were either left untreated (untreated; n = 20) or treated (n = 94) with an IVD containing 1.56 g of P4 (Cue‐mate, Vetoquinol, Brisbane). Animals in the untreated group on day 0 were separated and placed on adjacent pastures but were not administered any further treatments or yarded again until day 55. On day 0, apart from cows in the untreated group, cows were injected intramuscular (IM) with either 1 mL of 0.9% saline (Saline, n = 24, 15 heifers and 9 cows), 100 μg GnRH, (gonadorelin acetate, Cattle‐Mate; Randlab, Chipping Norton, NSW, Aust; n = 35, 25 heifers and 10 cows) or 250 μg GnRH (n = 35, 25 heifers and 10 cows). On day 5, IVDs were removed, aids for the detection of oestrus (Estrotect, Genetics Australia, Bacchus Marsh, Vic, Aust) were applied to the base of the tail and cloprostenol (0.5 mg; Juramate; Jurox, Rutherford, NSW, Aust) was administered IM at the time of removing IVDs and again 8.0 ± 0.11 h (mean ± SEM) later.

### Detection of oestrus

Between days 6 and 11, the treatment groups were monitored twice daily for at least 30 min at about 0600 and 1800 h for the onset of oestrus by observation of animal behaviour and for observed changes in aids used for the detection of oestrus. These aids were classified on a five‐point scale, based on the proportion of background colour that was visible and whether aids had been lost. The following scores were assigned: 0 = detector lost or 100% background colour visible, 1 = <100% to 75% visible, 2 = <75% to 50% visible, 3 = <50% to 25% visible, 4 = <25% to none of the background colour visible. Cows that were observed to stand while being mounted and cows with a score of ≤3 were classified as being in oestrus.

### Transrectal ovarian ultrasonography

Transrectal ultrasonography was performed on each cow using a 7.5 MHz, linear transducer (Mylab 5; Medical Plus Australia Pty Ltd, Tullamarine, VIC, Aust) on day −10. Cows in the Saline, GnRH‐100 and GnRH‐250 groups were examined again on days 5 and 7. Between days 5 and 11.5, once animals in the Saline, GnRH‐100 and GnRH‐250 groups were detected in oestrus, they were examined twice daily (about 0600 and 1800 h) with transrectal ultrasonography to detect ovulation. Animals that had not been detected in oestrus on days 9 and 11.5 were also examined with ultrasound on those days. On day −10 the presence or absence of a CL was recorded while at all other examinations ovarian maps were drawn to record the mean diameter ([maximum length + maximum width]/2) and location of the largest and second‐largest follicles and any corpora lutea present in each ovary. Electronic callipers were used to measure the diameters of ovarian structures. *Corpora lutea* were assumed to be induced by day 5 when no CL was observed to be present in an ovary on day 0 but a CL was visible in the same ovary on day 5 or if a second CL was visible on day 5 in an ovary when only one was observed in the same ovary on day 0.^8^ Cows without a CL detected on days −10 and 0 with transrectal ultrasound were classified as anovulatory by day 0. Those with a CL detected on either day −10 or 0 were classified as ovulatory by day 0.

### Blood sampling and hormone assays

Blood samples (approximately 10 mL) were collected from the coccygeal vein or artery into plain evacuated tubes, from a sub‐group of cows in the Saline (n = 15), GnRH‐100 (n = 15) and GnRH‐250 (n = 14) treatment groups on days 0 and 5. On day 0, samples were collected 1.35 ± 0.03 h (range: 0.95 to 1.85 h) after administration of saline or GnRH when an LH surge was expected^20^, ^30^, ^31^, ^32^, ^33^, ^34^, ^35^ and on day 5 samples were collected just prior to when inserts were removed. Blood samples were stored at room temperature for 1 hour then placed on ice and centrifuged at 3000 *g* within 4 h of collection. Serum was then separated and stored at −20°C until the time of assay. Concentrations of LH in serum samples collected on day 0 were measured by double‐antibody radioimmunonassay^36^ using Bovine LH antiserum (AFP‐192279; National Hormone and Peptide Program, Harbor‐UCLA Medical Center) and goat‐anti‐rabbit serum (Sac‐Cel, IDS, Bolton, UK). Bovine LH chloramine‐T‐based iodination was performed by Prosearch Australia (Malvern, VIC, Aust). The standard curve was prepared by serially diluting the LH antigen (bLH AFP‐11118B; National Hormone and Peptide Program, Harbor‐UCLA Medical Center, Torrance, CA, USA). The sensitivity of the assay was 0.2 ng/mL. The intra‐assay coefficient of variation for all sample duplicates was 3.1% and for sample quality controls of 2.2 and 9.3 pg/mL were 8.8% and 5.0%, respectively. Concentrations of P4 were measured in duplicate using a solid‐phase radioimmunoassay kit (IM1188, Immunotech, Prague, CZ).^37^ The sensitivity of the assay was 0.09 ng/mL. The intra‐assay variation for a plasma pool of 1.2 ng/mL was 7.7%.

### Hair sampling and determination of concentrations of cortisol

Hair was clipped on day 55 from the same animals and region that were clipped on day −10, dried, stored individually in airtight containers and protected from light until samples were prepared for assay. Sample preparation and analysis were based on the protocol by Stalder and Kirschbaum^38^ with minor modifications. Briefly, hair samples were washed three times for 3 min with 10 mL of isopropanol and left to dry for 12 h in a fume cupboard. Hair samples were then weighed and subsequently ground in a bead beater with 2.0 mm zirconium oxide beads (Bio Tools, Pty Ltd, Loganholme, QLD, Aust) in 2 mL micro vials (Eppendorf North America, Inc., USA) for 120 min. The samples were then incubated with 1.7 mL methanol and agitated at 200 rpm at room temperature for 23 h prior to centrifugation at 10,000 rpm for 5 min. A total of 1.1 mL supernatant was transferred into a new micro‐vial and evaporated under a constant stream of nitrogen gas while being maintained at 50°C until completely dry. Samples were reconstituted with 0.3 mL of assay buffer, vortexed for 15 s and immediately analysed with a commercial chemiluminescent immunoassay (Immulite1000 cortisol, Siemens Healthcare Diagnostic Products Ltd., Bayswater, VIC, Aust).^39^ The intra‐assay variation for a sample quality control of 30.1 ng/mL was 6.1%; the sensitivity was 1.7 ng/mL.

### Statistical analyses

Statistical analyses were performed using the statistical software IBM SPSS version 25 (SPSS Inc., Chicago, IL, USA). Analysis of variance was used to compare means. Factors included in models included treatment and age (heifer or cow) and the interaction between treatment and age. Post hoc comparison of differences between means was assessed using Tukey's test. Homogeneity of variance was assessed using Levene's test. Potential associations between concentrations of LH and P4 in serum on day 0 were assessed using both linear and inverse regression models. Multivariable logistic regression was used to determine the odds of inducing a new CL by day 5 and the odds of oestrus and ovulation between days 5 and 11.5. Factors considered in models included treatment, age (heifer or cow), body condition score, whether animals were ovulatory or anovulatory at the start of treatment, the diameter of the largest follicle and the number of follicles ≥8.5 mm on day 0, weight at the start of the study and relevant interaction terms. The presence of a CL on day 0 was also included in the assessment of the odds of inducing a new CL by day 5. In a separate model, the same statistical technique was used to investigate the potential association of induction of a new CL by day 5 with whether animals were detected in oestrus or not within 156 h of removal of IVDs. Age, whether animals were ovulatory or anovulatory and whether a new CL was induced were included in the model. A backwards‐stepwise method was used for variable selection although treatment was retained in every model, apart from the model used to determine whether induction of a new CL affected the oestrous response. Wald Chi‐square statistic was used for sequential variable elimination (at P = 0.10) and to test the significance of main effects (P = 0.05). Goodness‐of‐fit was assessed using the Hosmer Lemeshow test. Pearson's Chi‐Square test was used to compare the percentage of animals that were detected in oestrus between 48 and 72 h after removing intravaginal inserts. Regression analysis was used to compare the response and follicle diameters against treatments adjusting for animal age (heifer vs cows) and day of measurement (day 0, 5 and 7). To account for heterogeneity between animals linear mixed‐effects models were fitted^40^ where animal identification was used as a random effect. The packages lme4,^41^ lattice,^42^ and ggplot2^43^ were used for model fitting and visualization in software R 4.1.0.^44^ Data are presented with the mean ± standard error of the mean (SEM) unless otherwise stated, while P‐values ≤0.05 were considered significant and regarded as a tendency when 0.05 < P < 0.10.

## Results

### Exclusions

One heifer in the Saline‐treated group was removed from the study on day 5 due to poor temperament. Data from this animal was included up until day 5 but excluded thereafter. The concentration of LH for one heifer in the Saline‐treated group was omitted from the analysis of concentrations of LH as her serum concentration of LH was 6.4 ng/mL, which was >3 standard deviations (SD) from the mean concentration of the other animals in the same treatment group (0.51 ± 0.13 ng/mL; mean ± SD). This animal was presumably undergoing a spontaneous LH surge at the time of treatment. She was subsequently recorded as having a new CL by day 5. The percentage of animals within each age category, along with the mean weight, body condition and ovulatory status at the start of treatment, are given in Table 1.

**Table 1:** Age of animals, body condition, weight and ovulatory status at the start of treatment

Variable	Treatment								P‐values		
	Untreated		Saline		GnRH (100 μg)		GnRH (250 μg)				
Age	Heifer	Cow	Heifer	Cow	Heifer	Cow	Heifer	Cow	Treatment	Age	Treatment × age
n	13	7	15	9	25	10	25	10			
BCS (1–5)	3.5 ± 0.10	3.1 ± 0.14	3.5 ± 0.07	3.3 ± 0.14	3.7 ± 0.08	3.4 ± 0.11	3.5 ± 0.06	3.5 ± 0.17	0.451	0.050	0.450
Weight (kg)	374.7 ± 7.5	410.6 ± 14.5	376.1 ± 8.5	429.2 ± 14.1	382.0 ± 5.2	426.8 ± 18.7	371.1 ± 5.9	426.1 ± 16.5	0.392	0.001	0.816
Ovulatory day 0 (%)^a^	‐	‐	66.7 (10/15)	88.9 (8/9)	88.0 (22/25)	90.0 (9/10)	88.0 (22/25)	100.0 (10/10)	0.971	0.773	0.865
CL day 0 (%)	‐	‐	53.3 (8/15)	55.6 (5/9)	56.0 (14/25)	90.0 (9/10)	52.0 (13/25)	50.0 (5/10)	0.185	0.915	0.294
Follicles ≥8.5 mm day 0 (%)	‐	‐	53.3 (8/15)	66.7 (6/9)	64.0 (16/25)	80.0 (8/10)	40.0 (10/25)	80.0 (8/10)	0.742	0.045	0.593

^a^ Ovulatory status is determined by the visible presence of a CL in ovaries with transrectal ultrasonography on days −10 and 0. Animals in the untreated group were only examined once on day −10 so ovulatory status on day 0 was not determined.

BCS, body condition scored; CL, corpus luteum; GnRH, gonadotrophin releasing hormone.

In response to our objectives, we now discuss the effect of treatments (Saline vs GnRH‐100 and GnRH‐250 treatments) on three primary endpoints, oestrous and ovulatory responses, follicular development, concentrations of LH and P4 using logistic regression and repeated measures ANOVA, adjusting for age (heifers vs cows).

### Oestrous and ovulatory responses associated with treatment

The percentage of animals in which a new CL was induced between days 0 and 5 and the percentage of animals that were detected in oestrus and ovulated within 156 h of removing IVDs are listed in Table 2. Treatment did not significantly affect the odds that a CL was induced following treatment on day 0, but there was a positive association between the diameter of the largest follicle in the ovary and the odds of inducing a new CL (Table 3). The absence of a visible CL on day 0 was associated with greater odds that a new CL would be induced (Table 3). When the odds of inducing a new CL were compared only between animals treated with two dose rates of GnRH the odds of animals having a CL induced were not significantly affected by the dose of GnRH (P = 0.298) but was less in heifers compared to cows (8%, 4/50 vs 30%, 6/20, respectively; OR: 0.06, 95% CI 0.01 to 0.44; P = 0.006) and less in animals with a CL visible in the ovaries compared to those in which a CL was not visible (4.9%, 2/41 vs 27.6%, 8/29, respectively; OR: 27.9, 95% CI 2.72 to 285.5; P = 0.005).

**Table 2:** Ovulatory response to initial treatment with saline or two doses of GnRH and the oestrous and ovulatory response following removal of IVDs^a^

Variable	Treatment^a^		
	Saline	GnRH (100 μg)	GnRH (250 μg)
CL induced (%)	12.5 (3/24)	14.3 (5/35)	14.3 (5/35)
Detected in oestrus (%)	95.7 (22/23)	82.9 (29/35)	88.6 (31/35)
Ovulated (%)	87.0 (20/23)	82.9 (29/35)	77.1 (27/35)

^a^ Comparison between treatments for each variable P > 0.10; Table 3.

CL, corpus luteum; GnRH, gonadotrophin releasing hormone; IVD, intravaginal P4 releasing device.

**Table 3:** Analysis of odds ratio estimates for effects of treatment on binary variables after treatment with either saline or two doses of gonadotrophin releasing hormone (GnRH)

Dependent variable	Parameter	df	B	SE	χ^2^	Odds ratio	95% CI	P‐value^a^	Reference group
CL induced^b^	Saline^c^	1	−0.28	0.89	0.100	0.75	0.13 to 4.32	0.751	GnRH‐250^c^
	GnRH‐100^c^	1	−0.08	0.83	0.010	0.92	0.18 to 4.70	0.921	GnRH‐250
	Largest follicle day 0	1	0.40	0.16	6.59	1.50	1.10 to 2.04	0.010	‐
	No CL day 0	1	2.51	0.86	8.49	12.3	2.28 to 66.94	0.004	CL day 0
Detected in oestrus^d^	Saline	1	0.98	1.01	0.941	2.67	0.37 to 19.5	0.332	GnRH‐250
	GnRH‐100	1	−0.428	0.74	0.335	0.65	0.15 to 2.78	0.563	GnRH‐250
	Anovulatory^e^	1	−2.21	0.77	8.32	0.11	0.02 to 0.49	0.004	Ovulatory^e^
Ovulation detected^d^	Saline	1	1.03	0.80	1.66	2.81	0.59 to 13.5	0.197	GnRH‐250
	GnRH‐100	1	0.52	0.65	0.620	1.68	0.47 to 5.99	0.431	GnRH‐250
	Anovulatory	1	−2.28	0.71	10.37	0.10	0.03 to 0.41	0.001	Ovulatory

^a^ Statistical significance is based on Wald Chi‐Square test statistic.

^b^ Corpus luteum (CL) induced between days 0 and 5.

^c^ Animals were treated with saline, or 100 or 250 μg of GnRH on day 0.

^d^ Within 156 h of removing intravaginal devices.

^e^ Anovulatory—no CL detected on days −11 and 0, ovulatory—a CL detected on at least one of those days.

co‐efficient, CI, 95% confidence interval; df, degrees of freedom; SE, standard error.

The odds of animals being detected in oestrus within 156 h of removing IVDs were not significantly affected by treatment or age of animal but there was a significant difference in the odds of oestrus being detected in animals classified as ovulatory at the start of treatment compared to those that were classified as anovulatory (91.4%, 74/91 vs 61.5%, 8/13, respectively; OR: 0.11, 95% CI 0.02 to 0.49; P = 0.004; Table 3). Similarly, the odds of animals ovulating within the same time period were not significantly affected by treatment or age of animal but differed in the animals classified as ovulatory at the start of treatment compared to those that were classified as anovulatory (86.4%, 70/81 vs 46.2, 6/13, respectively; OR: 0.10, 95% CI 0.03 to 0.41; P = 0.001; Table 3). The mean interval to oestrus and ovulation after removal of IVDs did not vary significantly between treatments or between heifers and cows and no significant interaction between these two variables was found (Table 4). The mean interval to oestrus for all animals combined was 82.4 ± 2.5 h and the mean interval to ovulation was 111.3 ± 2.2 h. The variances in both the mean interval to oestrus and ovulation between the different treatment groups were also similar (P > 0.470). The odds that animals were detected in oestrus between 48 and 72 h after removing IVDs did not differ with treatment (OR: 0.39, 95% CI 0.11 to 1.38; P = 0.146) or age (OR: 1.62, 95% CI 0.62 to 4.23; P = 0.328) of animals. The percentage of animals detected in oestrus between 48 and 72 h of removing inserts for the saline, GnRH‐100 and GnRH‐250 treatments was 47.8% (11/23), 60.0% (21/35) and 37.1% (13/35), respectively (P = 0.160). The percentage of animals detected in oestrus that did and did not have a new CL induced by day 5 was 92.3% (12/13) and 87.5% (70/80), respectively (P = 0.619). Similarly, the odds of being detected in oestrus were not significantly affected by whether or not a new CL was induced (OR: 2.44, 95% CI 0.26 to 23.4; P = 0.438) with cycling status at the start of the study retained in the model as its effect was significant (OR: 5.93, 95% CI 1.36 to 25.8; P = 0.018).

**Table 4:** Characteristics of follicular development and interval to detection of oestrus and ovulation

Variable	Treatment			P‐values		
	Saline	GnRH (100 μg)	GnRH (250 μg)	Treatment	Age	Treatment x age
No. of corpora lutea day 0	0.54 ± 0.10	0.66 ± 0.08	0.51 ± 0.09	0.211	0.312	0.340
No. of corpora lutea day 5	0.58 ± 0.10	0.66 ± 0.09	0.60 ± 0.08	0.627	0.080	0.698
Interval to oestrus (h)^a^	83.5 ± 5.2	76.6 ± 4.1	87.1 ± 3.6	0.275	0.344	0.284
Interval to ovulation (h)^a^	111.0 ± 4.8	107.6 ± 3.6	115.6 ± 3.1	0.372	0.606	0.731
Diameter of the ovulatory follicle day 5^b^ (mm)	6.5 ± 0.46^A^	10.0 ± 0.28^B^	6.7 ± 0.35^A^	0.003	0.254	0.406
Diameter of ovulatory follicle (mm)	11.4 ± 0.31	11.8 ± 0.31	10.9 ± 0.23	0.087	0.029	0.638

^a^ Within 156 h of removing IVDs.

^b^ Diameter of the ovulatory follicle retrospectively traced to its diameter on day 5.

Means within rows with different uppercase superscript letters differ (P < 0.05).

### Characteristics of follicular development

**Figure 2:**
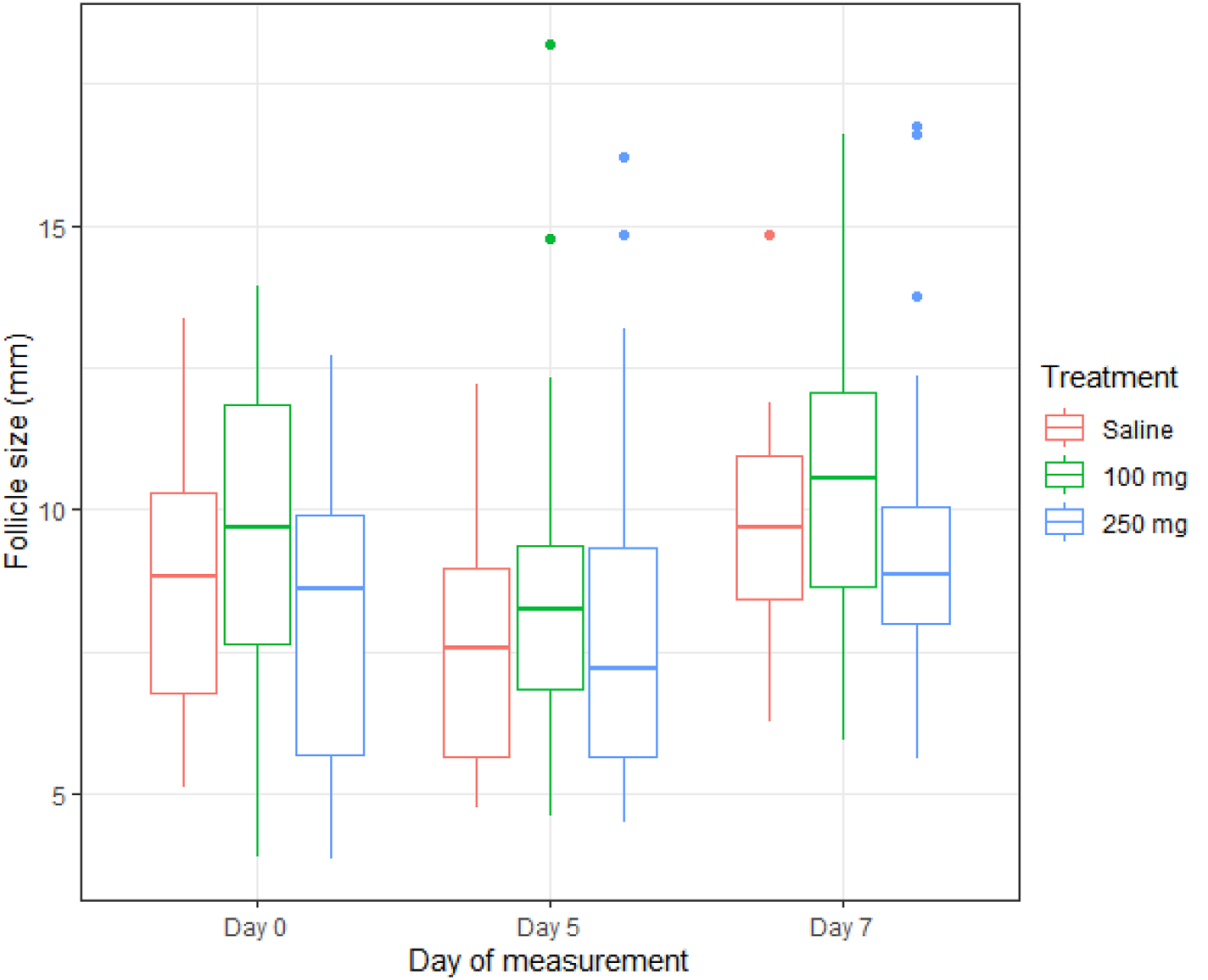
A boxplot illustrating the univariate distribution of maximum follicle diameter grouped by treatment and day of sampling (0, 5 and 7) in animals treated with saline (left boxplot), 100 μg of GnRH (middle box plot) or 250 μg of GnRH (right box plot) when concurrently treated with an intravaginal P4 releasing device from days 0 to 5 and cloprostenol (0.5 mg) twice on day 5. The box plot shows the 50th percentile (median, line within the box), 25th and 75th percentile (box), 10th and 90th percentiles (whiskers) and outliers (dots).

**Figure A1:**
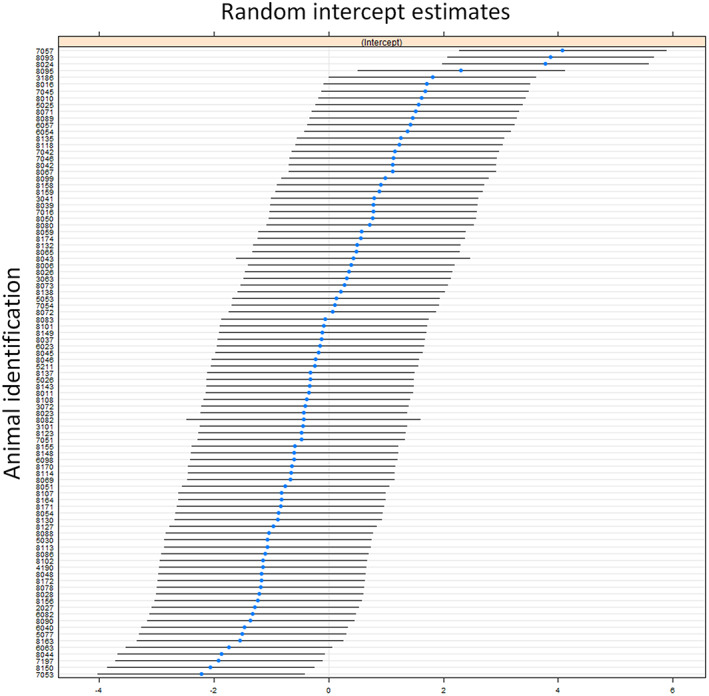
Random intercept estimates illustrating heterogeneity in baseline average follicle diameter (intercept) in a regression model comparing follicle diameter against treatment, adjusting for animal age (heifers vs cows) and day of measurement.

**Figure A2:**
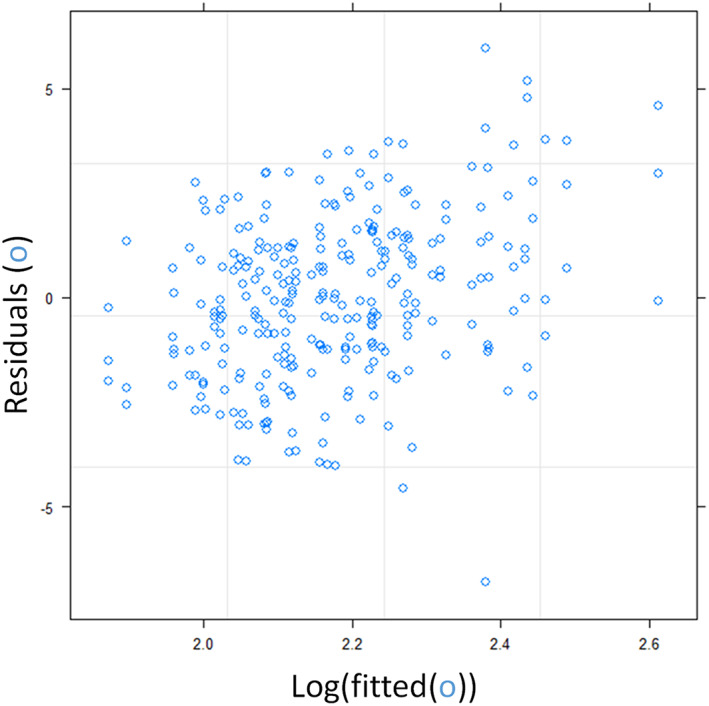
A scatterplot illustrating the residuals of the random effects model against the fitted values.

Boxplots illustrating the diameter of the largest follicle imaged within ovaries on days 0–7 are shown in Figure 2. Adjusting for heterogeneity between animals, no significant treatment effects were found for dosage levels of GnRH, 250 (P = 0.87) or 100 mg (P = 0.050), compared to the saline (control) group on the diameter of follicles between days 0 and 7. The diameter of the largest follicle in the ovary had a significant association with the age (heifer or cow) of animals (P < 0.001) but also the day of measurement. The diameter of the largest follicle imaged in the ovary on day 7 (P = 0.01) was significantly larger than day 0, irrespective of animal age and treatment. Further, we observed that compared to heifers, cows had a significantly larger follicle diameter on day 5 (P = 0.04), irrespective of other factors. Parameter estimates for the mixed‐effects model are shown in Table A1. We found significant heterogeneity of model intercepts between animals that were modelled using the random effect for animals (*σ* animal^ = 1.49, 95% CI 1.11 to 1.82). Figure A1 depicts the variation in intercepts (mean follicle diameter) by animal and Figure A2 depicts a scatterplot of the residuals of the random effects model against the fitted values.

The diameter of the ovulatory follicle was affected by age (P = 0.029) but not significantly by treatment (P = 0.087), or the interaction between the two (P = 0.638; Table 4). There was a tendency for the ovulatory follicle to have a greater diameter in the GnRH‐100 group compared to the GnRH‐250 group (Table 4; P < 0.10) although the variance in the means was homogenous (Levene's test, P = 0.451). The mean diameter of the ovulatory follicle was greater in cows compared to heifers (11.9 ± 0.35 mm vs 11.0 ± 0.17 mm, respectively; P = 0.029). The homogeneity of variance did not differ between any of the treatment groups for every variable examined regarding ovarian follicular development. The mean number of corpora lutea per animal on days 0 and 5 did not differ between treatments but there was a tendency for the number of corpora lutea on day 5 to be greater in the cows compared to the heifers (0.76 ± 0.09 vs 0.55 ± 0.06, respectively; P = 0.080; Table 4).

### Concentrations of LH and P4

**Figure 3:**
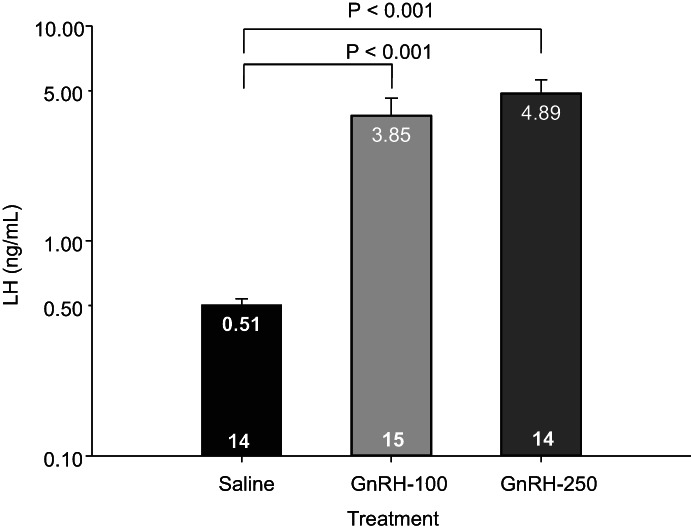
Mean ± SEM concentrations of LH 1.35 ± 0.03 h after administration of either saline, 100 or 250 μg of GnRH with concurrent administration of a P4 releasing intravaginal device. Note logarithmic (base 10) scale. Numbers at the base of bars represent the number of animals.

Mean concentrations of LH were transformed (log 10) before analysis as variances were not homogeneous (P = 0.006). Mean concentrations of LH, analysed from the blood sample taken 1.35 ± 0.03 h after administration of treatment, were significantly affected by treatment (P ˂ 0.001) but not by age (P = 0.947) or the interaction between treatment and age (P = 0.242). Mean concentrations of LH were less in the animals treated with saline compared to the animals treated with either dose of GnRH (P < 0.001) but did not differ significantly between the animals treated with the different doses of GnRH (Figure 3). Due to the negative association found between the presence of a CL on day 0 and the odds that a new CL was induced we also examined the mean concentrations of LH on day 0 in the GnRH‐treated animals with and without the visible presence of a CL on day 0. Mean concentrations of LH did not, however, differ significantly between GnRH‐treated animals with and without a CL visible in the ovaries (3.67 ± 0.16 pg/mL vs 4.99 ± 1.01 pg/mL, P = 0.966).

Concentrations of P4 in serum on day 0 were available from 42 animals and were consistent ≥3.0 ng/mL. The mean concentration of P4 in serum on day 0 (15.2 ± 1.25 ng/mL) did not differ significantly between treatments (P = 0.751), age (P = 0.566) and the interaction between the two (P = 0.423). Similarly, on day 5, the mean concentration of P4 (9.98 ± 0.85 ng/mL) did not differ significantly between treatments (P = 0.299), age (P = 0.113) and the interaction between the two (P = 0.068). Mean concentrations of P4 in serum on day 0 were less in animals treated with GnRH in which a new CL was recorded to occur by day 5 compared to those in which a new CL was not recorded on day 5 (8.25 ± 2.23 vs 17.75 ± 1.71 ng/mL, respectively; P = 0.011). Animals treated with GnRH with a CL visible in the ovaries on day 0 tended to have greater mean concentrations of P4 in serum after administering the IVD on day 0 compared to animals in which a CL was not visible (18. 9 ± 2.63 vs 13.0 ± 1.77 ng/mL, respectively; P = 0.068). No significant linear (r^2^ = 0.017, P = 0.522) or inverse (r^2^ = 0.022, P = 0.441) relationship was found between concentrations of LH and P4 in serum on day 0.

### Concentrations of cortisol in hair

**Figure 4:**
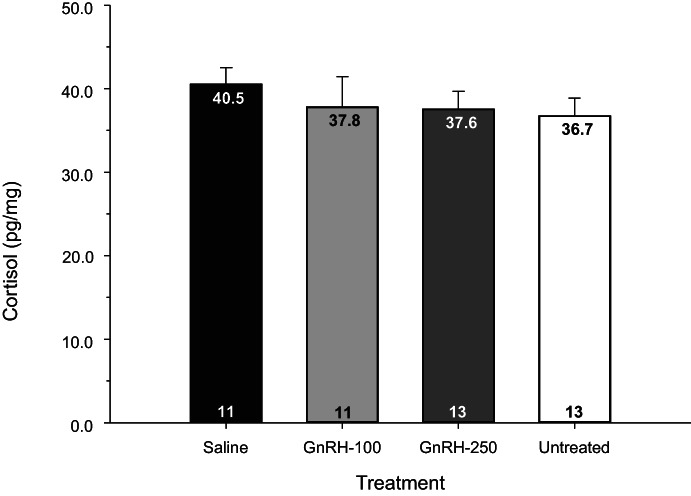
Mean ± SEM concentrations of cortisol in hair samples collected 55 days after animals were either left untreated or were treated with either saline or 100 or 250 μg of GnRH as part of a 5‐day, progestin‐based protocol to synchronise oestrus. All except the untreated animals were also monitored for the onset of oestrus and ovulation with transrectal ultrasonography between days 5 and 11. Numbers at the base of bars represent the number of animals.

The mean concentration of cortisol in hair samples collected 55 days after the start of treatment was not significantly affected by treatment (P = 0.83; Figure 4) or age (P = 0.24) and no significant interaction between these two variables was found (P = 0.65).

## Discussion

In general, about 50% or more of cows and heifers can fail to ovulate following administration of GnRH at the start of commencing a protocol to synchronise oestrus and ovulation which can affect fertility to a timed AI.^4^, ^6^, ^8^, ^9^, ^11^, ^13^, ^14^, ^15^, ^20^, ^45^ In this study there was even a substantially lower response to the first treatment with GnRH compared to most previously reported studies with only 14.3% of animals treated with either dose of GnRH with a visible new CL 5 days after treatment. Increasing the dose of GnRH has increased LH responses to treatment and ovulation rates in some studies, but in the present study, LH responses and the odds of inducing a new CL after administration of GnRH were not increased by using 250 μg compared to 100 μg of GnRH as gonadorelin. We, therefore, reject the hypothesis that increasing the dose of gonadorelin administered from 100 to 250 μg would increase the odds of inducing a new CL in *B*. *indicus* cows and heifers when administered at the start of treatment with an IVD. Stress responses can also be associated with the handling of animals and management interventions. Analysis of HCC in animals administered no treatments compared to those that were treated and monitored regularly was not found to differ significantly. This suggests that elevated stress responses were not associated with the treatment and monitoring of animals in this study or could not be detected using the methodology used in this study.

In the present study, a significant increase in mean concentrations of LH was recorded using both doses of GnRH compared to the saline‐treated animals, although no significant difference was found in the mean concentrations of LH in animals administered different doses of GnRH. A greater concentration of LH after administration of GnRH compared to saline was expected and has been previously reported.^30^, ^46^ Souza et al.^20^ found a doubling in the maximal concentration of LH and an 80% increase in the area under the LH curve when 100 compared to 50 μg of various analogues of GnRH were administered to dairy cows. Giordano et al.^21^ reported that dairy cows administered 200 μg compared to 100 μg of GnRH had a greater LH peak and area under the curve in cows with both high and low circulating concentrations of P4. An increase in ovulatory response from 58% to 67% was also demonstrated by the same research group when administering 200 μg compared to 100 μg of GnRH.^9^ Chenault et al.^30^ demonstrated a progressive increase in peak concentrations of LH as the dose of gonadorelin administered to Holstein heifers increased from 50 to 500 μg. Thus a greater peak concentration of LH was expected in the animals in this study administered 250 μg compared to 100 μg of GnRH. Maximum concentrations of LH have been consistently recorded between 1 and 2 h after administration of GnRH.^20^, ^30^, ^31^, ^32^, ^33^, ^34^, ^35^ The timing of blood collection in this study should, therefore, have coincided with the expected peak concentration of LH after administration of GnRH. We only collected one blood sample to reduce the stress load on animals, but it is possible that the LH profiles differed over time in animals administered different doses of GnRH, or that the timing of sampling may not have precisely coincided with when peak concentrations of LH occurred. Nevertheless, the biological response in terms of the number of new corpora lutea induced was identical.

One possible reason for a lack of difference in mean concentrations of LH between animals administered the two different doses of GnRH in the present study could be related to high mean concentrations of P4 in serum that were found around the time of the expected LH peak in animals treated with IVDs (15.2 ± 1.25 ng/mL). High concentrations of P4 in serum were likely due to the rapid increase in circulating concentrations of P4 that occurs following administration of IVDs.^47^, ^48^ High concentrations of P4 may indirectly decrease the number of GnRH receptors in the anterior pituitary by suppressing GnRH secretion.^49^, ^50^ This could reduce the sensitivity of the pituitary to the administration of GnRH, thereby reducing the pituitary release of LH. This may also have contributed to the lack of difference in mean LH response between the different dose rates of GnRH that were administered.

Concentrations of P4 in plasma have been negatively associated with the magnitude of a GnRH‐induced LH surge in cattle.^21^, ^31^, ^32^, ^33^ We, however, found no significant association between concentrations of P4 and concentrations of LH. As blood samples were collected soon after administration of GnRH and insertion of an IVD there were no animals with low concentrations of P4 at the time of sampling and less opportunity to compare responses between animals with low and high concentrations of P4. This highlights the need for future studies examining LH and ovulatory responses for a blood sample to be collected before treatments are administered. We did record lesser mean concentrations of P4 in serum on day 0 after administering an IVD in animals in which a new CL was induced by day 5 compared to those in which no new CL was induced. In animals treated with GnRH on day 0, we also recorded a negative association between the presence of a CL on day 0 and the odds of a new CL being recorded by day 5. This supports the results of other studies that collectively suggest that elevated serum concentrations of P4, especially in the presence of a CL, could inhibit the ovulatory response following administration of GnRH in cattle administered GnRH concurrently with an IVD.^33^, ^51^, ^52^

Mean concentrations of P4 on day 0 in animals treated with GnRH that lacked a visible CL (17.75 ± 1.71 ng/mL) were recorded as being somewhat greater than what is routinely achieved in the peripheral circulation of cattle following treatment with an IVD for up to 8 days where mean concentrations usually of between 2 and 6 ng/mL have been recorded.^53^, ^54^, ^55^ The timing of collection of blood samples soon after administration of IVDs could explain the relatively high mean concentrations of P4 recorded at that time.^54^, ^55^ Although the sensitivity of detecting corpora lutea with transrectal ultrasonography is high (89.4%)^56^ it is still possible that some functional corpora lutea were not observed with the techniques used in this study which would have also contributed to elevated concentrations of P4 measured on day 0.

The diameter of an ovarian follicle at the time of GnRH treatment affects the odds that it will ovulate^15^, ^57^ which is related to the presence of LH receptors on granulosa cells.^58^ Gimenes et al.,^59^ also reported that ovulatory capacity progressively developed after follicles reached 7 mm in diameter in *B*. *indicus* heifers with 33.3% (3/9), 80.0% (8/10) and 90% (9/10) of heifers ovulating after administration of porcine LH with emerging follicle diameters of 7.0–8.4 mm, 8.5–10.0 mm and >10 mm, respectively. On day 0 the percentage of animals with at least one follicle ≥8.5 mm ranged between 40 and 80% but did not differ significantly between treatments. We included both the number of follicles ≥8.5 mm and the diameter of the largest follicle in the ovary in the logistic regression models. Adjusting for these variables in the model, no significant difference was found in the odds of inducing a new CL after administration of any of the treatments. The poor ovulatory response in *B*. *indicus* females in this study and especially heifers could be due to an inadequate proportion of animals with follicles that developed to an ovulatory capacity at the time of administering GnRH or that follicles had an inadequate number or receptivity to LH. Further study is needed to examine the LH response to administration of GnRH and to examine LH receptor numbers at different stages of follicular development in *B*. *indicus* cattle to explore physiological reasons for sometimes poor ovulatory responses to treatment.

A greater percentage of cows compared to heifers developed a new CL after administration of GnRH in this study (30%, 6/20 vs 8%, 4/50, respectively) which is consistent with the findings of others in dairy cattle.^6^ Ovulation rates to GnRH in heifers in other studies have generally been greater than in this study ranging between 25 and 56% with the stage of the oestrous cycle and the diameter and maturity of ovarian follicles at the time of administration being the major determinants of the ovulatory response.^60^, ^61^, ^62^

The implications of our findings of induction of a low number of corpora lutea after administration of GnRH, particularly in *B*. *indicus* heifers, are that there may be little advantage in administering GnRH analogue gonadorelin when using a progestin‐based protocol to synchronise oestrus. Lamb et al.^63^ found that pregnancy rates in beef heifers treated with a 7‐day duration of treatment with P4 that received GnRH at the time of insertion of a P4 releasing insert (55%, 571/1034) were similar to heifers that did not receive GnRH at insertion (52%, 541/1041). Similarly, other studies have not found a significant decrease in pregnancy rates when GnRH was omitted at the time of insertion of progestin devices during a 5‐day CIDR‐CO‐Synch protocol in dairy heifers^61^, ^62^, ^63^, ^64^ and yearling beef heifers.^62^ Kasimanickam et al.,^65^ however, found that when using a 5‐day, CIDR‐CO‐Synch protocol in beef heifers, the administration of GnRH at the time of insertion of IVDs, improved pregnancy rates to AI by 8%. Helguera et al.^66^ also demonstrated an increase in pregnancy rate of 13.8% in dairy heifers that were acyclic at the start of the program when GnRH was included at the start of a 5‐day CoSynch plus IVD treatment. Failure to induce a new CL in most heifers administered GnRH in this study and the similar synchrony of oestrus compared to heifers administered saline suggest that there would be no benefit in administering GnRH to *B*. *indicus* heifers treated with a 5‐day, progestin‐based protocol. The limited response in cows (30%) also suggests that any improvement in synchrony may be small. Given the greater odds of inducing a new CL observed in this study in animals with larger follicles and lacking a visible CL in the ovaries at the time of administering GnRH, redesigning protocols to remove CLs and increase the diameter of the largest follicle in the ovary at the time of administering GnRH may improve ovulation rates to the administration of GnRH. Further changes to the protocol may also require an increase in the number of interventions required prior to AI which may be impractical in an extensive management beef herd.

In this study, the poor apparent ovulatory response after administering GnRH and a likely failure to synchronise a new follicular wave would have contributed to variability in the pattern of follicular development between days 0 and 5. As a result, we did not record differences in the mean intervals to and synchrony of oestrus and ovulation between treatments. Further work is needed to develop methods for synchronizing oestrus with GnRH that result in induction of ovulation and synchronise follicular growth patterns and yield more precise synchrony of oestrus and ovulation. In addition, fertility responses were not evaluated in this study and should also be considered, along with an examination of responses in larger numbers of animals to further evaluate responses to GnRH when using P4‐based protocols.

One potential concern when using a 5‐day treatment phase between administering GnRH on day 0 and a luteolytic treatment on day 5 is that failure of luteolysis could occur in any new corpora lutea that is induced from treatment on day 0. Incomplete luteolysis after administering prostaglandin F2 alpha or an analogue can occur when heifers and cows are treated less than 5 and 7 days, respectively after oestrus.^67^, ^68^ In this and other studies that have utilised a 5‐day treatment protocol two injections of prostaglandin F2 alpha or an analogue are administered within 24 h of removal of IVDs to help ensure that luteolysis occurs. In this study, a similar number of animals with and without new corpora lutea induced on day 5 were detected in oestrus supporting the efficacy of this protocol. However, only a relatively small number of animals with newly induced corpora lutea were available in this study for comparison so further study examining oestrous responses in animals with newly formed corpora lutea and luteolytic treatments 5 days after administering GnRH may be warranted.

One objective of the present study was to determine if there was a stress response that could be detected in hair samples collected from *B*. *indicus* cattle in association with treatments used to synchronise oestrus. We did not detect significant differences in HCC between any of the treatment groups and, therefore, we reject our hypothesis that greater stress responses would be associated with the administration of treatments used to synchronise oestrus and monitoring of *B*. *indicus* females compared to untreated control animals. The main stressors in the current study were likely to be frequent handling and close contact with humans, stress associated with the treatments that were imposed, blood sampling and rectal examinations. Previous studies have provided some evidence that IVDs can induce mild inflammatory reactions,^69^, ^70^ vaginitis^22^, ^23^ and, depending on the type of insert administered, increased frequency of straining and defaecating.^71^ The lack of significant differences in mean HCCs between treatments may have been due to the cattle becoming acclimatised to the frequent handling and human contact during or prior to the study or could be related to facilities enabling animals to be handled in a relatively calm manner.^72^, ^73^ It could also be that the period of the investigation when treatment and monitoring occurred (days 0–11) was too short or the stress load imposed on animals was too low to induce differences in HCC between treatments by the time samples were collected. Heimburge et al.^27^ suggested that HCC is a useful marker for long‐term stress but not for short, single or scarce events. Further research is needed to examine more acute behavioural and physiological responses that are used to assess stress responses^72^ to confirm the findings of this current study that treatments and interventions applied to cattle resulted in no detectable increase in stress loading compared to untreated cattle.

## Conclusions

Increasing the dose of GnRH from 100 to 250 μg at the time of insertion of an IVD in *B*. *indicus* females did not increase mean concentrations of LH 1.35 ± 0.03 h after treatment or increase the odds of inducing a new CL. Ovulatory responses and the synchrony of oestrus and ovulation were also similar to the administration of saline. Odds of inducing a new CL after treatment with GnRH were greater in cows compared to heifers and in animals with larger follicles and in those that lacked a CL at the time of administration. This result raises the question as to whether the use of GnRH, as gonadorelin at the start of a 5‐day, progestin‐based protocol to synchronise oestrus and ovulation is necessary. Fertility responses were not evaluated in this study and should also be considered, along with the examination of responses in larger numbers of animals to further evaluate responses to GnRH when using progestin‐based protocols. The magnitude of stress responses associated with P4‐based protocols to synchronise oestrus appeared similar to untreated cattle and suggests that any stress associated with treatments and experimental interventions was relatively mild, of short duration or could not be detected by measuring HCC. Further research is needed to find alternatives to using gonadorelin to synchronise follicular wave emergence or to find other means of optimising responses to GnRH in *B*. *indicus* cattle.

## Conflicts of interest and sources of funding

S. Edwards is an employee of Vetoquinol Australia who distributes Cue‐Mate devices and Cattle‐Mate (GnRH) injections. She was not involved in data acquisition or the analyses of results. All other authors declare no conflict of interest. Vetoquinol Australia provided funding for pharmaceuticals, assays and materials used in the study. Financial support for M. Abdallah was provided by Australia Awards Africa and James Cook University also provided financial aid for the study.

## APPENDIX A

**Table A1:** Model estimates of fixed and interaction effects for linear mixed effects modelling

Fixed effects	Estimate	SE	df	t‐value	P	2.5% CI	97.5% CI
(Intercept)	5.64	0.85	182.79	6.61	0.00	3.99	7.29
Age (1: Heifer)	2.15	0.55	212.85	3.89	0.00	1.08	3.22
Treatment (100 mg)	0.99	0.50	90.52	1.98	0.05	0.02	1.96
Treatment (250 mg)	−0.08	0.50	90.76	−0.16	0.87	−1.05	0.89
Day 5	0.99	0.86	182.48	1.15	0.25	−0.69	2.67
Day 7	2.28	0.87	183.19	2.63	0.01	0.59	3.97
Age:Day 5	−1.30	0.62	182.48	−2.10	0.04	−2.52	−0.09
Age:Day 7	−0.90	0.62	182.82	−1.44	0.15	−2.11	0.32

CI, confidence limit; df, degrees of freedom; SE, standard error.
